# Supplementary material for: Airborne Isolation Cardiac Arrest: A Simulation Program for Interdisciplinary Code Blue Team Training
Source: MedEdPORTAL. 2022 Jan 14;18:11213. doi: 10.15766/mep_2374-8265.11213 (PMC8758800; doi:10.15766/mep_2374-8265.11213)
Supplement: Supplementary file 1 — Protocol Diagram.docxTraining Video.mp4Simulation Case Template.docxSimulation Images.pdfAction Priorities.docxSimulation Script.docxSurvey.docx [file mep_2374-8265.11213-s001.zip › G. Survey.docx]

**Airborne Isolation Code Blue Simulation Survey**

The following asks you to assess your confidence in knowledge, skills and safety relating to airborne isolation code blue.

1. **What is your title?**
2. Acute Care Unit RN
3. Intensive Care Unit RN
4. STAT RN
5. Intern (PGY 1)
6. Resident (PGY 2-3)
7. Medical student
8. Hospitalist
9. Nursing student
10. Patient care technician
11. Advanced practice provider
12. Respiratory therapist
13. Other (please explain)
14. **Did you receive any of the following training for airborne isolation code blue prior to the session today?**
15. Watched protocol video
16. Read protocol flyer
17. Training during nursing huddle
18. Participated in real life airborne isolation code blue
19. No prior training but I have heard of UWMC’s airborne isolation code protocol
20. No prior training and I have never heard of UWMC’s airborne isolation code protocol
21. Other (please explain)
22. **AFTER today’s training, I am able to identify:**

|  | Strongly disagree | Disagree | Neutral | Agree | Strongly agree |
| --- | --- | --- | --- | --- | --- |
| Which code team members should enter the patient’s room |  |  |  |  |  |
| When I need to wear airborne isolation PPE |  |  |  |  |  |
| Where to acquire appropriate PPE |  |  |  |  |  |

1. **BEFORE today’s training, I was able to identify:**

|  | Strongly disagree | Disagree | Neutral | Agree | Strongly agree |
| --- | --- | --- | --- | --- | --- |
| Which code team members should enter the patient’s room |  |  |  |  |  |
| When I need to wear airborne isolation PPE |  |  |  |  |  |
| Where to acquire appropriate PPE |  |  |  |  |  |

1. **AFTER today’s training, I feel prepared to:**

|  | Strongly disagree | Disagree | Neutral | Agree | Strongly agree |
| --- | --- | --- | --- | --- | --- |
| Identify my role on the code team |  |  |  |  |  |
| Communicate effectively in a two team environment |  |  |  |  |  |
| Transfer items into and out of the code room |  |  |  |  |  |

1. **BEFORE today’s training, I felt prepared to:**

|  | Strongly disagree | Disagree | Neutral | Agree | Strongly agree |
| --- | --- | --- | --- | --- | --- |
| Identify my role on the code team |  |  |  |  |  |
| Communicate effectively in a two team environment |  |  |  |  |  |
| Transfer items into and out of the code room |  |  |  |  |  |

1. **AFTER today’s training, I am confident I will be able to:**

|  | Strongly disagree | Disagree | Neutral | Agree | Strongly agree |
| --- | --- | --- | --- | --- | --- |
| Provide quality care to my patient |  |  |  |  |  |
| Protect myself from high risk exposure |  |  |  |  |  |
| Protect my colleagues from high risk exposure |  |  |  |  |  |

1. **BEFORE today’s training, I was confident I would be able to:**

|  | Strongly disagree | Disagree | Neutral | Agree | Strongly agree |
| --- | --- | --- | --- | --- | --- |
| Provide quality care to my patient |  |  |  |  |  |
| Protect myself from high risk exposure |  |  |  |  |  |
| Protect my colleagues from high risk exposure |  |  |  |  |  |

1. **What feedback or concerns do you have regarding airborne isolation code blue?**
