# Supplementary material for: Airborne Isolation Cardiac Arrest: A Simulation Program for Interdisciplinary Code Blue Team Training
Source: MedEdPORTAL. 2022 Jan 14;18:11213. doi: 10.15766/mep_2374-8265.11213 (PMC8758800; doi:10.15766/mep_2374-8265.11213)
Supplement: Supplementary file 1 — Protocol Diagram.docxTraining Video.mp4Simulation Case Template.docxSimulation Images.pdfAction Priorities.docxSimulation Script.docxSurvey.docx [file mep_2374-8265.11213-s001.zip › A. Protocol Diagram.docx]

| **Airborne Isolation Code: Inside Team Roles  (print and laminate)**  Inside Code Team is ESSENTIAL personnel only. Others remain outside unless called to enter by the Outside Code Team Leader. | | | 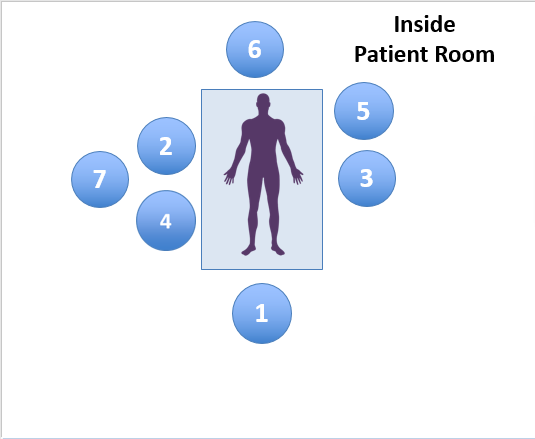 |
| --- | --- | --- | --- |
| 1 | Inside Code Leader | First or most senior MD/Advanced practice provider (APP)  Direct Resuscitation  Communicate with Outside Code Team Leader to obtain needed equipment, meds, or personnel |  |
| 2 | Bedside RN | Provide History  Compressor  Assist in vascular access, lab draws, and equipment transfer |  |
| 3 | ICU RN (STAT) | Rhythm monitoring and defibrillation/pacing  Access: IO or other as needed, draw labs |  |
| 4 | Med RN (usually code team ICU RN; may be filled by Acute Care RN) | Administer meds and fluids  Facilitate meds transfer from Outside to Inside  Ensure closed loop communication to Inside Code Leader and Outside Recorder RN  Assist STAT as needed with access, blood draw  Backup compressor |  |
| 5 | Respiratory Therapy | Secure Airway  Bag valve mask w/ expiratory HEPA viral filter or set ventilator |  |
| 6 | Anesthesia (if intubation needed) | First/most senior responding anesthesiologist only. One assistant if absolutely necessary  Establish airway  Assist with procedures per Inside Code Leader  Backup compressor after airway established | |
| 7 | Additional RN/ Compressor (usually ICU RN from Code Team; may be filled by Acute Care RN | While not doing compressions: perform pulse checks, hand off equipment, labs and meds to/from Transfer RN | |
| 8 | Surgical Airway or another surgical emergency | First / most senior responding surgeon only. One assistant if absolutely necessary | |

| **Airborne Isolation Code: Outside Team Roles  (print and laminate)**  Outside Team members should line hallway walls to facilitate movement of supplies, people | | | 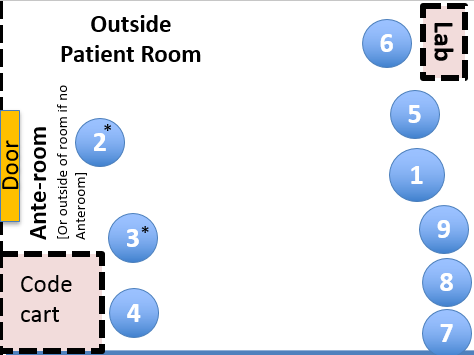 |
| --- | --- | --- | --- |
| 1 | Outside Code Leader | First qualified MD/APP to arrive after Inside Team Leader  Organize Outside code team members  Assist communication between Inside and Outside Teams Act on Inside Team Leader requests   - Ensure adequate Compressors. Replace when: Compressor has had three cycles or is fatigued - CPR quality is suffering |  |
| 2 | Transfer RN   - Wears Droplet PPE* - Stands in anteroom or warm zone | Get Stuff in and out of room  Hand in all equipment/meds, ensure Inside Team aware.  Accept specimens from Inside, clean and hand to Lab |  |
| 3 | Trained PPE* Observer   - Stands outside of warm zone | Get People in and out of room  Observe donning/doffing procedures |  |
| 4 | Pharmacist | Hand meds and supplies to Transfer RN to go into room  Calculate medication dosages, prepare meds |  |
| 5 | Recorder RN | Document code activities and times  Complete room entry log  Supplies walkie talkies |  |
| 6 | Lab | Remain Outside the room  Manage lab specimens as in a typical code |  |
| 7 | Spiritual Care, Social Work | Ensure family contacted by phone or in person  Assist with family communication | |
| 8,9 | Runners & relief compressors | Assist in obtaining supplies and handing to Transfer RN  Be ready to enter room to assist per Outside Team Leader | |
| UWMC Airborne Code Blue Diagram. *PPE (Personal protective equipment)  *Images author owned* | | | |
