# Supplementary material for: Airborne Isolation Cardiac Arrest: A Simulation Program for Interdisciplinary Code Blue Team Training
Source: MedEdPORTAL. 2022 Jan 14;18:11213. doi: 10.15766/mep_2374-8265.11213 (PMC8758800; doi:10.15766/mep_2374-8265.11213)
Supplement: Supplementary file 1 — Protocol Diagram.docxTraining Video.mp4Simulation Case Template.docxSimulation Images.pdfAction Priorities.docxSimulation Script.docxSurvey.docx [file mep_2374-8265.11213-s001.zip › C. Simulation Case Template.docx]

| **Appendix C: *MedEdPORTAL* Simulation Case Template**  **SIMULATION CASE TITLE: Airborne Isolation Code Blue**  **AUTHORS: Alexandra C. Collis MD, Andrew P. Wescott MD, PhD, Sheryl Greco MN, RN, Nicole Solvang RN, BSN, CCRN, Joshua Lee MD, Amy E. Morris MD**  **LEARNER AUDIENCE: Physician code leaders (residents, fellows, advanced practice providers, faculty), acute and critical care unit nurses, and other code blue responders (rapid response nurses, respiratory therapy, anesthesia, medical technicians, medical students, pharmacy). Ideal number of learners: 6-8. Minimal number required 5. Maximum number that can be accommodated by script: 10** | |
| --- | --- |
| **PATIENT NAME:** Taylor/Cameron/Danny  **PATIENT AGE:** 37  **CHIEF COMPLAINT:** Cardiac arrest  **PHYSICAL SETTING:** Acute care or ICU patient room | |
|  | |
| **Brief narrative description of case** | CC – “Patient found unresponsive and pulseless”   1. HPI/Hospital Course:   (COVID-19 airborne precautions) Taylor is a 37 year-old with no past medical history who initially presented with the chief complaint of shortness of breath and cough. They were diagnosed in the ED with COVID-19 pneumonia and were found to be hypoxemic requiring 4L Nasal Canula (NC). They were admitted to the acute care floor (or ICU) 4 days ago for further management. They were started on steroids and Remdesivir but their oxygen requirement increased to 6L nasal cannula. The patient is on airborne isolation precautions.  The bedside nurse enters the room alone in airborne isolation precautions to evaluate the patient and finds that the patient is unconscious. The nurse shouts the patient’s name with no response and is not able to feel a pulse so they press the code blue call button and begin chest compressions.   1. Alternate HPI/Hospital Course:   (Other airborne precautions) Danny is a 37 year-old with no past medical history who presented with the chief complaint of hemoptysis. They were admitted to the acute care floor (or ICU) and diagnosed with pulmonary Mycobacterium tuberculosis. They were placed on airborne isolation precautions.  Four days after admission, the bedside nurse enters the room alone in airborne isolation precautions to evaluate the patient and finds that the patient is unconscious. The RN shouts the patients name with no response and is not able to feel a pulse so they press the code blue call button and begin chest compressions   1. Alternate HPI/Hospital Course:   (Not already on airborne precautions): Cameron is a 37 year-old with no past medical history initially presented with the chief complaint of R arm pain and swelling. They were diagnosed today in the ED with acute kidney injury and were admitted to the acute care floor (or ICU). The patient denied any shortness of breath, cough, loss of smell or taste, or recent known exposure to COVID-19. COVID-19 screening is performed and the result is pending. On arrival to the floor they are placed on regular precautions given no high risk symptoms or signs of COVID-19. Within 2 hours they suddenly develop a new oxygen requirement to 4L NC O2. The beside nurse enters the room to evaluate the patient and they are not responsive. The nurse yells the patients name and is not able to feel a pulse so hits the code blue button and begins chest compressions. The patient is not on airborne isolation precautions but hospital policy dictates airborne isolation for all patients with unknown COVID-19 status.  Case Progression / Narrative:  After bedside nurse identifies cardiac arrest, calls a code blue, and begins chest compressions, the outside team is alerted and key code blue members arrive to the room.  Cases 1 and 2: Inside code team members (second compressor, ICU nurse, inside code team leader, anesthesia, respiratory therapy, or per hospital protocol) are directed by simulation script to don airborne isolation PPE and enter the room.  Case 3: Once second compressor enters the code room and takes over chest compressions, the bedside nurse should exit the room, don airborne isolation PEE, and reenter the room to resume their role as 1^st^ compressor.    Outside code team members (transfer nurse, trained PPE donning and doffing observer, outside code leader, recorder nurse, pharmacist, additional ICU nurse, or per hospital protocol) should remain outside the code room and not don airborne isolation PPE. Outside code leader is prompted by simulation script to identify inside code team members to make sure that all needed personnel are present. They will then pass walkie-talkies and initial code blue medications and supplies into the room.  Inside code team leader will utilize standard ACLS to run inside code and narrate code events to outside code leader and recorder nurse. Outside code leader will update inside code leader at the time of key events such as next pulse check/defibrillation. They will also facilitate getting essential supplies and equipment into the room by directing these items to the transfer nurse who will pass them in.  Inside instructor will provide the pulse check rhythm. Pulse check one is PEA, pulse check two is V fib and inside code leader should direct defibrillation to be performed, and the patient will regain a pulse at pulse check three. |
| **Primary Learning Objectives** | 1. Identify which code blue response team members should enter an airborne isolation code room (“Inside Team”) and which team members remain outside (“Outside Team”). *(Patient care, systems-based practice)* 2. Describe the role of each participant in an airborne isolation code blue. *(Patient care, systems-based practice)* 3. Describe where to find airborne isolation personal protective equipment (PPE). *(Patient care, Systems based practice)* 4. Demonstrate use of walkie-talkies to communicate between inside and outside code teams. *(Systems based practice, interpersonal skills and communication)* 5. Demonstrate transport of items into and out of the code room while observing safety protocols. *(Systems based practice, interpersonal skills and communication)* |
| **Critical Actions** | Outside code leader:   - Confirm all needed personnel are inside the code room - Confirm all needed equipment is inside the code room - Confirm closed loop communication with inside code team using walkie-talkie - Alert inside code members to time of next pulse check/rhythm check   Inside code team leader:   - Don airborne PPE prior to entering room - Confirm closed loop communication with outside team using walkie-talkie - Narrate code script prompts to outside team using walkie-talkies   Transfer RN:   - Pass walkie-talkies into room - Pass defibrillator and initial medications into room - Transfer labs out of room, clean vials per floor protocol - Alert inside code team at time of any transfer by knocking on door or visual cues if applicable - Recognize when inside team needs to transfer items out of room - Demonstrate item transfer without entering room - Ensure door is closed when not actively transferring items   Bedside nurse:   - Demonstrate coordinated transfer of chest compressions with second responder - (If not initially in airborne) Demonstrate exiting room and donning airborne PPE before reentering - Recognize when outside team needs to transfer items into the room - Accept transferred items from transfer nurse when applicable   Second responder:   - Bring initial unit supply of airborne PPE to the entry area of the room - Don airborne PPE prior to entering room - Do not bring PPE bucket into code room - Recognize when outside team needs to transfer items into the room - Accept transferred items from transfer nurse when applicable - Alert transfer nurse of lab vial transfer through knocking or visual cues if applicable |
| **Learner Preparation or Prework** | Pre-session learning materials are provided prior to the session for asynchronous learning and review: video training module, nursing huddles, educational flyers. Immediately prior to the case, the instructor leads a 10 minute pre-brief to talk-through the protocol steps and roles. Each role is discussed in detail and learners are encouraged to ask questions. The script framework is also explained to learners. |

| Initial Presentation | | | |
| --- | --- | --- | --- |
| **Initial vital signs** | *Pulseless and unresponsive* | | |
| **Overall Setting and Appearance** | *CPR manikin in bed in typical patient room on the unit* | | |
| **Standardized Participants (and their roles in the room at case start**) | *Instructor: gives HPI and initial cue that patient has been found pulseless by bedside RN.*  *ICU nurse standardized participant: outside room in hallway, waiting to enter until code is called as per script.* | | |
| **HPI** | *All information volunteered by instructor:*  *Taylor is a 37 year-old with no past medical history initially presented with the chief complaint of shortness of breath and cough. They were diagnosed in the ED with COVID-19 pneumonia and were found to be hypoxic requiring 4L Nasal Canula. They were admitted to the acute care floor (or ICU) 4 days ago for further management. They were started on steroids and Remdesivir but their oxygen requirement increased to 6L nasal cannula. The patient is on airborne isolation precautions.*  *The bedside nurse just entered the room to evaluate the patient and finds that the patient is unconscious and without a pulse.* | | |
| **Past Medical/Surgical History** | **Medications** | **Allergies** | **Family History** |
| No past medical history | Dexamethasone 6mg IV daily, Remdesivir 100mg IV daily, Albuterol MDI, 2 puffs every 6 hours PRN  Alternative HPI #2: isoniazid 300mg PO daily, rifampin 600mg PO daily, pyrazinamide 1g PO daily, ethambutol 800mg PO daily  Alternative HPI #3: cephalexin 500mg every 6 hours | No known drug allergies | History of type 1 Diabetes Mellites in mother, otherwise no known family history |
| **Physical Examination** | | | |
| **General** | Unresponsive and pulseless | | |
| **HEENT** | Pupils equal, round and reactive to light, oropharynx clear | | |
| **Neck** | Supple | | |
| **Lungs** | No respirations | | |
| **Cardiovascular** | Pulseless, no lower extremity edema | | |
| **Abdomen** | Soft, nontender, nondistended, no hepatosplenomegaly | | |
| **Neurological** | Unresponsive | | |
| **Skin** | Cool to the touch, no rashes or wounds | | |
| **GU** | Not examined | | |
| **Psychiatric** | Unable to assess as patient is unconscious | | |

| Instructor Notes - Changes and CASE Branch Points | | |
| --- | --- | --- |
| **Intervention / Time point** | **Change in Case** | **Additional Information** |
| *Beginning of case* | *Patient is unresponsive and pulseless* | *Bedside RN recognizes cardiac arrest, hits code blue button, and begins chest compressions* |
| *(If not initially in airborne isolation PPE)* | *2^nd^ compressor enters the room* | *Bedside RN should leave the room, don airborne PPE, and then reenter to resume compressions* |
| *Code blue alarm sounds on the unit* |  | *Second compressor should call a code blue, bring the unit supply of airborne PPE to the room, don, and enter* |
| *First provider (physician, APP) arrives to room, asks outside RNs if there is already a code leader in the room* |  | *No inside code leader, so first provider should don airborne PPE and enter* |
| *Second provider arrives to the room and asks if there is already a code leader inside the room* | *Transfer nurse alerts provider there is already an inside code leader* | *Second provider identifies themselves as the outside code leader* |
| *1^st^ Pulse check performed* | *ICU reports no pulse present and rhythm PEA* | *Inside code leader should direct team to restart chest compressions* |
| *Stat nurse alerts code leader that more epinephrine is needed* |  | *Inside code leader should ask outside code leader for more epinephrine; outside code leader should ask transfer RN to pass in epinephrine* |
| *2^nd^ Pulse check performed* | *ICU nurse reports no pulse present and rhythm V fib* | *Inside code leader should direct ICU nurse to defibrillate patient and then to resume chest compressions* |
| *3^rd^ Pulse check performed* | *ICU nurse reports patient has a pulse* | *Inside code leader should alert outside code leader. Should ask for 1L IV fluids, norepinephrine. Outside code leader should ask transfer RN to pass items in. Case Ends.* |

**Ideal Scenario Flow**

The bedside nurse recognizes that the patient is in cardiac arrest, presses the code button and begins chest compressions. A second nurse on the unit hears the code bell, calls the operator to trigger a code blue, brings the PPE supply bucket to the code room, dons airborne PPE and enters the room. Bedside nurse and the second nurse alternate compressions. Meanwhile, a third nurse arrives outside at the room and identifies themselves as the “trained observer” to don and doff anyone that enters the room. A fourth nurse also arrives at the room and volunteers to be the transfer nurse outside the room. The first code leader arrives to the room and asks the nurses if there is already a physician inside the room. The nurses say no, so the first code leader dons airborne PPE, enters the room, and begins to lead the code as the Inside code leader using standard ACLS protocol. A second code leader, an ICU nurse, and a recorder nurse (played by instructor and bringing walkie-talkies) arrive at the room. The code leader asks if there is already a code leader inside. The nurses tell them there is, so the second code leader identifies themselves as the Outside code leader. The Outside code leader asks the transfer nurse if all essential personnel as well as a backboard, defibrillator, and HEPA filter are already in the room. The transfer nurse responds that there is no defibrillator in the room. The Outside code leader directs the ICU nurse to carry an initial supply of medications, the defibrillator, and the walkie-talkies into the room. Inside code leader receives the walkie-talkie and updates Outside code leader on the status of the code. The recorder nurse is next to the Outside code leader, overhears the exchange and documents events thus far. Inside the room, the ICU nurse applies the defibrillator and the Inside code leader pauses for a pulse check. The assistant instructor in the room informs Inside code leader there is no pulse and the rhythm is PEA. Inside code leader orders team to resume chest compressions. Outside code leader follows script to ask if more medications are needed and transfer nurse passes in epinephrine. Inside team passes labs out to the transfer nurse, and a third compressor is requested and sent into the room. Recorder alerts Outside code leader at time of pulse check; this is relayed by walkie-talkie to Inside code leader. At second pulse check the patient is pulseless, in V fib, and undergoes defibrillation. At third pulse check the patient has a pulse. IV fluids and vasopressors are requested from the Outside team via walkie-talkie. Once these items are transferred into the room by the transfer nurse, the simulation ends.

**Anticipated Management Mistakes**

1. *Outside supply of personal protective equipment is carried into the code room: The 2^nd^ responder nurse often brought the PPE supply bucket into the airborne isolation code room with them, making the PPE contaminated and unusable. Even after we educated learners on this during the introduction, this continued to happen. To prevent this occurrence during a real code blue, this is included as a critical action and we allowed learners to make this mistake during the simulation and then paused to immediately point it out to the whole group so that it would be memorable.*
2. *Challenges with communication: many learners found communicating with walkie-talkies difficult. Often, Inside code leaders continuously held down the walkie-talkie button when narrating the inside code events, such that Outside code leader could not ask questions or offer advice. We instituted an independent walkie-talkie training session for residents prior to simulation during which we covered essential use of these devices: ensure the walkie-talkies are on the same channel, use maximal volume, and release the talk button when not speaking. We also encouraged practice of backup methods of communication such as writing on the glass door or window, and using room phones. This was also included as a critical action and the simulation should continue until learners are able to demonstrate effective communication.*
3. *Inside code team loses their place in the script: the inside code team sometimes had difficulty figuring out where the simulation was in the script. To help with this we used an inside assistant to prompt the inside team to key events. Learners were also directed to use the script as a loose guideline and to follow prompts from others in the simulation.*
