# Supplementary material for: Airborne Isolation Cardiac Arrest: A Simulation Program for Interdisciplinary Code Blue Team Training
Source: MedEdPORTAL. 2022 Jan 14;18:11213. doi: 10.15766/mep_2374-8265.11213 (PMC8758800; doi:10.15766/mep_2374-8265.11213)
Supplement: Supplementary file 1 — Protocol Diagram.docxTraining Video.mp4Simulation Case Template.docxSimulation Images.pdfAction Priorities.docxSimulation Script.docxSurvey.docx [file mep_2374-8265.11213-s001.zip › D. Simulation Images.pdf]

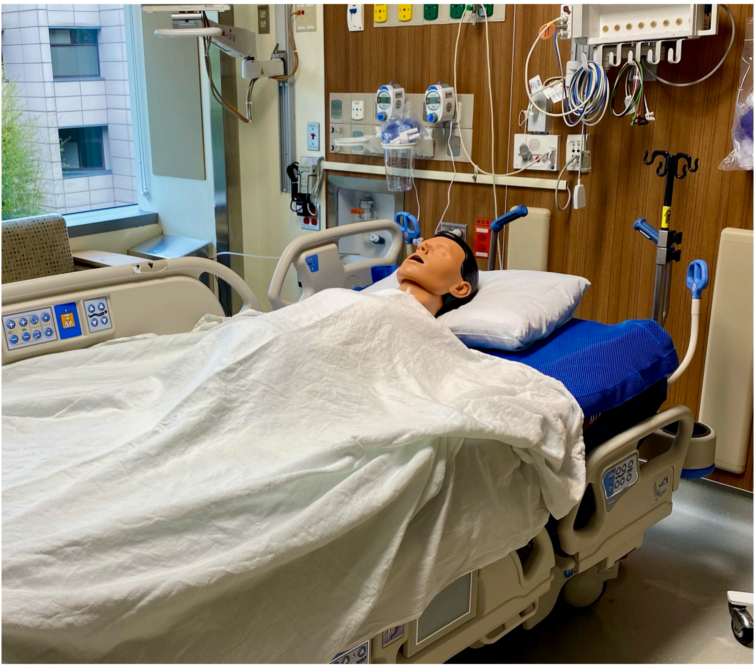

Simulation set up: CPR manikin positioned in unit bed. Image author owned.

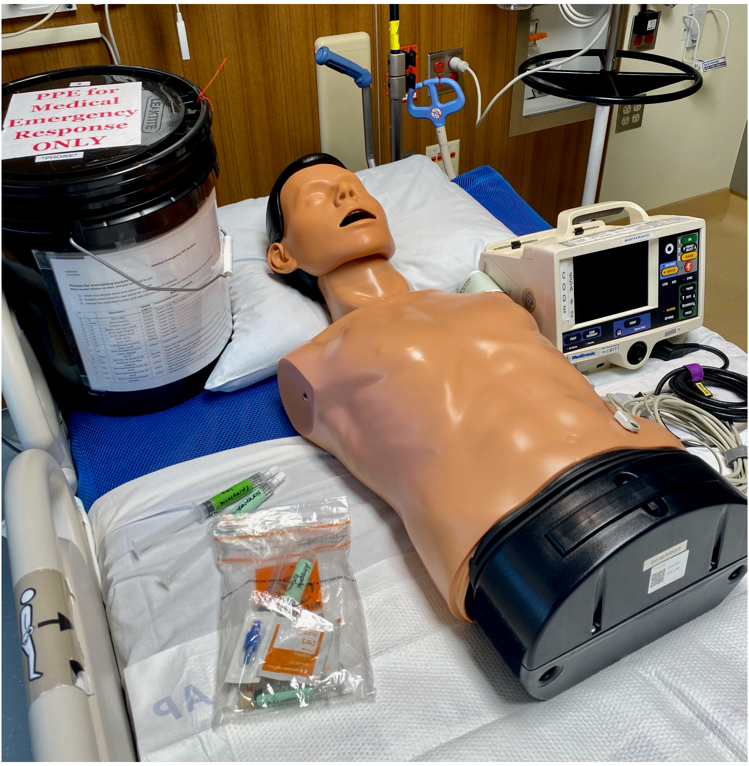

Equipment: PPE bucket, CPR manikin, defibrillator, and simulated medications (epinephrine, amiodarone). Image author owned.

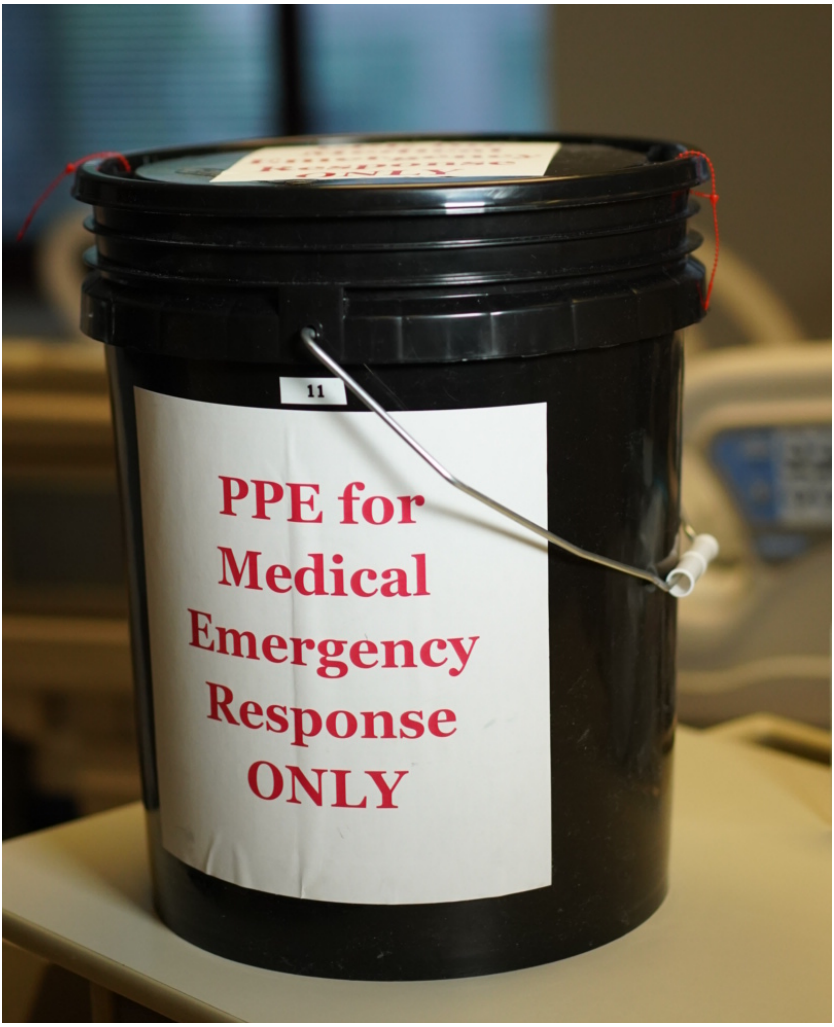

UWMC personal protective equipment, "PPE bucket." Image author owned.

"PPE Bucket" Contents	
Quantity	Contents
1	Hepa filter
1	Hand sanitizer bottle
7	Isolation gowns
8	Masks with face shields
5	N-95 1860 Regular
5	N-95 1860S Small
3	N-95 1870+
3	N-95 1870
1	N-95 Duckbill Kimberly Clark, Small
1	N-95 Duckbill Kimberly Clark, Regular
1	Phone
4	Security tie beaded red
1	Airborne Code Blue Diagram
1	Donning/Doffing Guide for N-95
1	Donning/Doffing Guide for PAPR
1	Code leader Action Priorities/Supply Checklist

Simulation Equipment	
1	CPR manikin
1	PPE bucket
10	Reusable contact isolation gowns
2	Walkie talkies (1 set)
2	Simulated epinephrine syringes
2	Simulated amiodarone syringes
1L	Simulated Lactated Ringers
1	Backboard
1	CPR stool
1	Defibrillator
1	Crash cart

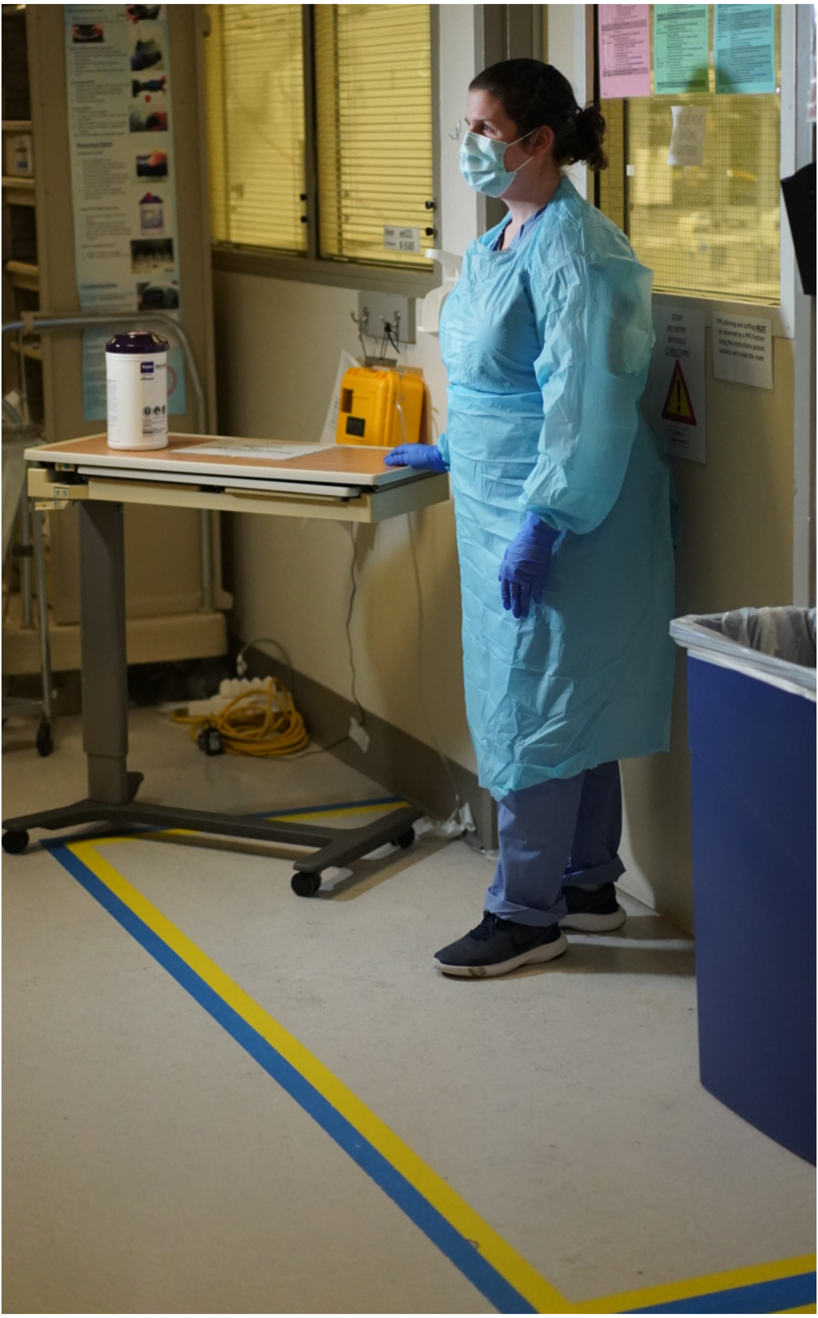

"Transfer RN" in warm zone demarcated by tape outside of the code room. Transfer RN is a nurse responsible for facilitating transfer of essential items into and out of the code room. When available, the transfer nurse is positioned in the anteroom, when not, they remain in the warm zone as pictured here. Image author owned.
