# Supplementary material for: Airborne Isolation Cardiac Arrest: A Simulation Program for Interdisciplinary Code Blue Team Training
Source: MedEdPORTAL. 2022 Jan 14;18:11213. doi: 10.15766/mep_2374-8265.11213 (PMC8758800; doi:10.15766/mep_2374-8265.11213)
Supplement: Supplementary file 1 — Protocol Diagram.docxTraining Video.mp4Simulation Case Template.docxSimulation Images.pdfAction Priorities.docxSimulation Script.docxSurvey.docx [file mep_2374-8265.11213-s001.zip › E. Action Priorities.docx]

**Action Priorities (print and laminate this page)**

1. **Identify Inside Code Team members and Trained Observer(s)** to facilitate room entry. Emergency PPE Bucket in unit med room contains supplied for 8-10 responders.

Prioritize: Compressor to relieve first responder

STAT RN

Med RN

Inside Code Leader

Respiratory Therapy

1. **Ensure immediate equipment** goes in with the first Inside Team members:

- Defibrillator and pads
- Backboard
- Initial meds: Epi, atropine, bicarb, saline, pressure bag/tubing
- IO drill/catheter (if no central access)

1. **Identify Outside Code Team members: especially Transfer RN**
2. **Additional equipment:** RN and Pharmacist to load clear bags with:

- Meds: epinephrine, amiodarone vials, lidocaine, mag vials, bicarb
- NS flush syringes, 10mL syringes for drawing up meds
- Additional NS liter bag(s) with pressure bad and tubing
- ABG syringes/lab tubes

1. **Establish communication with Inside Code Team**. Options:

- Walkie-talkie
- Marker on paper/whiteboard against window/door glass
- Speakerphone (in PPE bucket)
- Other phones such as spectralink phones
