# Supplementary material for: Airborne Isolation Cardiac Arrest: A Simulation Program for Interdisciplinary Code Blue Team Training
Source: MedEdPORTAL. 2022 Jan 14;18:11213. doi: 10.15766/mep_2374-8265.11213 (PMC8758800; doi:10.15766/mep_2374-8265.11213)
Supplement: Supplementary file 1 — Protocol Diagram.docxTraining Video.mp4Simulation Case Template.docxSimulation Images.pdfAction Priorities.docxSimulation Script.docxSurvey.docx [file mep_2374-8265.11213-s001.zip › F. Simulation Script.docx]

***Airborne isolation Code Blue Simulation Script:***

**Instructor:** The Bedside RN is alone in the patient’s room. The patient suddenly becomes hypotensive and loses a pulse. The Bedside RN recognizes the patient’s cardiac arrest, hits the code button and begins chest compressions.

***Bedside RN: (Hit code button and then start compressions.)***

**Instructor**: A second nurse on the unit hears the code bell and calls a code blue. They get the PPE bucket from the medication room and bring it to room.

**Second Compressor: *(Call code, get PPE bucket, and go to room.)*** I called the code and have the PPE bucket.

***Second Compressor: (Don airborne PPE and enter the room.)***

***Bedside RN and Second compressor: (Alternate compressor/bagging.)***

**Instructor:** Meanwhile, a third RN comes to the room to be the trained observer. A forth RN also arrives to be the transfer nurse.

**Trained Observer: *(Assume role outside room, ready to don and doff.)*** I will be the Trained Observer.

**Transfer RN:** I can be the Transfer Nurse.

**Instructor:** The first MD, Inside Code Leader arrives.

**Inside Code Leader: *(To RNs outside room)*** Is there already a code leader in the room?

**Transfer RN:**  There isn’t a code leader in the room yet.

***Inside Code Leader:***  ***(Identify that there is not yet an inside code leader, don airborne precautions, and enter. Begin to lead code.)***

**Instructor:** A Second MD, Outside Code Leader now arrives to the room. A STAT nurse and Recorder RN also arrive, bringing walkie-talkies.

**Outside Code Leader**: ***(To RN outside room)*** Who is already in the room?

**Transfer RN:** There are 2 compressors and an Inside Code Leader already in the room. I am the Transfer Nurse.

**Outside Code Leader:** We still need respiratory therapy and anesthesia inside the room. Do we have a defibrillator, backboard, and HEPA filter in the room?

**Transfer RN:** We have a HEPA filter and backboard, but we do not have the defibrillator. Anesthesia and respiratory therapy are on their way.

**Outside Code Leader:** STAT RN, please bring the defibrillator and meds bag into the room with you

***Pharmacist: (Pass meds bag to STAT RN.)***

**STAT RN:** I’ll bring the defibrillator and meds in with me. ***(Don and bring defibrillator and meds into the room.)***

**Outside Code Leader*: (Get walkie-talkies from recorder, turn on and check channel.)*** Transfer RN, please pass the walkie-talkies into the room along with additional meds.

**Transfer RN*: (Pass in walkie-talkies and meds.)***

**STAT RN: (*Once enter room, set up defibrillator. Ask inside code leader if they want a pulse check/rhythm check.)*** I do not feel a pulse and the rhythm is V fib. Do you want to charge now and plan to shock?

**STAT RN: (*After shock delivered)*** Do you want a dose of epi? I think that it’s time. **(*Pretend to give epi.)***

**Inside Code Leader: (*Into walkie-talkie)*** Outside code leader, can you hear me? Over.

**Outside Code Leader:** I can hear you. Where are you in the code? Over.

**Inside Code Leader:** (***Into walkie-talkie***) We have completed 2 rounds of chest compressions. We just did a rhythm check and the rhythm was V fib, we delivered the first shock and are resuming chest compressions now. 1 dose of epinephrine was given. Over.

**Outside Code Leader:** Do you need any more meds or another compressor? Over.

**Inside Code Leader:** We need one more dose of epi. We do not need another compressor. Over.

***Pharmacy:* *(Gives epi to Transfer RN.)***

***Transfer RN: (Pass epi in.)***

**Outside Code Leader: (*Into walkie-talkie)*** Have you thought about getting labs? Over.

**Inside Code Leader:** We will draw labs now. Over.

***STAT RN:*** (***Collect labs.*** ***Knock on door when labs ready, pass to transfer RN to clean.)***

**Recorder RN:** There are 30 seconds until next pulse check

**Outside Code Leader:** You have 30 seconds until next pulse check. Over.

**Recorder RN:** It’s time for a pulse check

**Outside Code Leader:** Perform pulse check now. Over.

**Inside Code Leader:** Performing pulse check now. Over. STAT RN, please perform a pulse check now.

**STAT RN:** I do not have a pulse, the rhythm, is PEA.

**Inside Code Leader:** Please resume chest compressions. ***(Into walkie-talkie)*** We have completed a pulse check and the patient does not have a pulse. The rhythm was PEA and we have resumed chest compressions. Over.

**Outside Code Leader: (*Into walkie-talkie)*** Do you need another compressor?

**STAT nurse: *(To code leader)*** The compressor looks like they are getting tired and may need to switch out.

**Inside Code Leader:** Compressors, please switch compressions during next pause for rescue breaths. ***(Into walkie-talkie)*** We will need a third compressor in the room. Over.

**Outside Code Leader:** ***(Assign 3^rd^ compressor role.)*** Sending in another compressor. Over.

***3^rd^ compressor: (Don and enter room.)***

**Recorder RN:** There are 30 seconds until next pulse check.

**Outside Code Leader:** You have 30 seconds until next pulse check. Over.

**Recorder RN:** It is now time for a pulse check.

**Outside Code Leader:**  It is now time for a pulse check. Over.

**Inside Code Leader:** Performing pulse check now. Over. STAT RN, please perform a pulse check.

**STAT RN:** I have a pulse.

**Inside Code Leader: *(Into walkie-talkie)*** The patient has a pulse. We are recycling the blood pressure. Can you pass in 1 L of LR and norepinephrine. We are collecting more labs and will send them out. Over.

**Outside Code Leader:** Sending in 1L LR and norepinephrine. The charge nurse is calling MICU. Over.

***Pharmacy:* *(Hand norepinephrine and 1L LR to Transfer RN.)***

***Transfer RN:* *(Pass meds in and labs out.)***

**Instructor:**  We will now start post arrest management. Those no longer needed in the room should doff and exit the room.

**Primary roles and descriptors:** Roles and assignments may be modified as needed for participant population and training environment. Instructor may read any roles that are unfilled to facilitate simulation.

**Simulation instructor(s)**

- **Instructor:** Supplies background information and prompts. Helps outside code team stay on script if there is confusion

May also play the following roles as needed:

- **Recorder RN:** Updates outside code leader to times of key events as per script. Supplies walkie-talkies
- **Pharmacy:** Supplies prop medication bags
- **STAT RN** (if no STAT nurse participating in training session may be played by instructor or assistant, see below for details)

**Resident (or other provider) roles:**

- ***Inside Code Leader:** Identifies they are first code leader to arrive and enters room. Leads inside team per standard ACLS guidelines based on prompts from *Outside Code Leader* and *STAT RN.* Narrates code and communicates with *Outside Code Leader* using walkie-talkies
- **Outside Code leader:** Identifies they are the second code leader to respond. Assumed role outside of room, follows “action priorities” to ensure essential equipment, medications, and team members are inside the room. Uses walkie-talkies to communicate with *Inside Code Leader* and alert them to timed events supplied by *Recorder RN*

**Unit nursing roles:** (may be assigned to medical technician is applicable)

- ***Bedside RN:** First unit nurse to identify cardiac arrest, alternates compressions**
- ***Second Compressor**: 2^nd^ unit nurse who will bring PPE bucket to room, enter, and alternate compressions with bedside RN**
- **Trained Observer:** Observes and assists other members don and doff PPE.
- **Transfer RN:** Assists with communication, transfer of items into and out of the room, and acts as gate keeper. Positioned outside code room door, in anteroom, or in “hot zone” outside room.
- ***3^rd^ Compressor:** (optional) Enters room when other compressors are fatigued, alternates compressions**

**STAT nursing roles:**

- ***STAT RN:** Enters room and alerts inside code team to key simulation events (see script). Administers prop medications and lab draws. Alerts code team of rhythm and pulse as per script. Helps inside code team stay on script if there is confusion

**Optional roles:**

- **Respiratory therapy**
- **Anesthesiologist**
- Additional simulation observers

*Don airborne PPE before entering code room. Reusable gowns are worn to represent “airborne PPE” to avoid use of real PPE

** Compressors are directed to put down script while performing chest compressions and are directed by STAT nurse on any further actions to take

***(Actions to perform italicized in script)***

Walkie-talkie training before simulation exercise is highly recommended

Supplies/Equipment:

- Half manikin (optional)
- Prop medications
- Prop lab tubes
- Defibrillator (pads not required)
- Walkie-talkie set
- 5-8 Reusable gowns (can substitute other marker of “airborne PPE”)
- Unoccupied patient room with hospital bed

**Alternative scenario:**

If patient not already in airborne precautions: bedside nurse should leave room after 2^nd^ compression arrives, change into airborne precautions and reenter the room to resume compressions.
