# Supplementary material for: Airborne Isolation Cardiac Arrest: A Simulation Program for Interdisciplinary Code Blue Team Training
Source: MedEdPORTAL. 2022 Jan 14;18:11213. doi: 10.15766/mep_2374-8265.11213 (PMC8758800; doi:10.15766/mep_2374-8265.11213)
Supplement: Supplementary file 1 — Protocol Diagram.docxTraining Video.mp4Simulation Case Template.docxSimulation Images.pdfAction Priorities.docxSimulation Script.docxSurvey.docx [file mep_2374-8265.11213-s001.zip › Educational_Summary_Report_11213.pdf]

MedEdPORTAL® | The AAMC Journal of Teaching and Learning Resources

Original Publication Open Access

Airborne Isolation Cardiac Arrest: A Simulation Program for Interdisciplinary Code Blue Team Training

Alexandra C. Collis, MD*, Andrew P. Wescott, MD, PhD, Sheryl Greco, MN, RN, Nicole Solvang, RN, BSN, CCRN, Joshua Lee, MD, Amy E. Morris, MD

*Corresponding author: acollis@uw.edu

Abstract

Introduction: In-hospital cardiac arrest in patients with COVID-19 presents significant challenges to health care teams. Airborne precautions can delay patient care, place providers at high risk of virus exposure, and exacerbate an already stressful environment. Within the constraints of an ongoing pandemic, an efficient educational program is required to prepare health care teams for airborne isolation code blue. **Methods:** This simulation was conducted in a room on the target unit using a CPR manikin to represent the patient. A "talk-through walk-through" scripted simulation directed learners (internal medicine residents, unit nurses, and other code blue responders) through a resuscitation using an airborne isolation code blue protocol. Key scripted events prompted role identification, communication, and item transfer. Learners self-assessed their airborne isolation code blue knowledge and skills and their confidence in providing quality care while maintaining safety using a pre-/posttraining 5-point Likert-scale survey. **Results:** We trained 100 participants over a 5-month period, with 65 participants surveyed (43 respondents; 16 residents, 22 nurses). Following training, participants had a statistically significant ($p < .001$) increase in percentage selecting agree/strongly agree for all statements related to knowledge and skills specific to airborne isolation code blue protocol, as well as confidence in providing care while keeping themselves and their colleagues safe. **Discussion:** Our simulation program allowed a small number of educators to feasibly train a large number of learners, let learners practice required skills, and improved learners' self-assessed knowledge, skills, and confidence regarding quality and safety of care.

Keywords:
COVID-19, Simulation, Interdisciplinary Medicine, Hospital Medicine, Resuscitation, Critical Care Medicine

Educational Objectives

By the end of this simulation, learners will be able to:

1. Identify which code blue response team members should enter an airborne isolation code room (inside team) and which team members should remain outside (outside team).
2. Describe the role of each participant in an airborne isolation code blue.
3. Describe where to find airborne isolation personal protective equipment.
4. Demonstrate use of walkie-talkies to communicate between inside and outside teams.
5. Demonstrate transport of items into and out of the code room while observing safety protocols.

Citation:
Collis AC, Wescott AP, Greco S, Solvang N, Lee J, Morris AE. Airborne isolation cardiac arrest: a simulation program for interdisciplinary code blue team training. *MedEdPORTAL*. 2022;18:11213.
https://doi.org/10.15766/mep_2374-8265.11213

Introduction

In-hospital cardiac arrest in patients with known or suspected COVID-19 carries a risk of virus transmission. Despite the arrival of successful vaccines, new variants are spreading worldwide, and there are reports of serious illness even among vaccinated individuals.[1] In addition, vaccination rates remain low in many vulnerable communities, and health care workers are not themselves universally vaccinated.[2] These concerns create a substantial emotional burden for health care teams trying to provide optimal care while protecting themselves and their colleagues.[3,4] Additionally, resident code leaders in training hospitals rotate through numerous clinical settings and must rapidly adapt to each unit while navigating the challenges of airborne personal protective equipment (PPE), which can compromise communication and lead to delayed CPR initiation and first defibrillation.[5-8]

Copyright © 2022 Collis et al. This is an open-access publication distributed under the terms of the Creative Commons Attribution license.

In response to these issues, many hospitals have designed airborne isolation code blue protocols.[8-11] The American Heart Association (AHA) suggests a two-team model with fewer personnel inside the code room to decrease direct exposure and ration the use of PPE.[8,12] Without adequate education, however, such a protocol may present more risk than benefit.[7] Our hospital initially distributed our AHA-based COVID-19 code blue protocol through several modalities, including training at nursing huddles, flyers, and a training video (Appendices A and B). However, ongoing challenges identified during real-life airborne isolation code blue events prompted the development of an interdisciplinary simulation curriculum.

Simulation is a well-established and effective means of education for code blue training,[13] particularly in preparing for situational variations with which health care teams may have less experience.[14-17] At the start of the COVID-19 pandemic, several groups successfully used high-fidelity simulation to train staff for airborne isolation code blue.[18-20] Unfortunately, this method of simulation requires significant resources, including personnel, time, and expensive equipment, that are limited in many institutions.[21,22] Among the few published protocols using alternative approaches, one interim report showed improved confidence and preparedness for COVID-19 cardiac arrest using a low-fidelity model, yet continued to rely on a time- and personnel-intensive simulation structure.[23] Prior to the pandemic, a scripted simulation structure was used for cardiac arrest training and resulted in improvements in team dynamics comparable to traditional high-fidelity simulation.[24] Similarly, Lee, Chen, Tosif, and Graham describe a “talk-through walk-through” approach for process development, testing, and education of endovascular clot retrieval in COVID-19 patients.[25] This simulation model, adapted from engineering task analysis techniques, consists of a single, two-part session with a brief verbal outline to mentally rehearse a procedure, followed by a walk-through to facilitate physical practice.[25] To our knowledge, there is no current literature on the use of these techniques for COVID-19 cardiac arrest and no published resources for implementing such a program.

During the COVID-19 pandemic, our hospital needed cardiac arrest training that was low cost, required few resources, and was quick to broadly implement. We initiated an in situ, interdisciplinary simulation program using a brief talk-through walk-through scripted isolation code blue event to enforce key learning objectives specific to airborne isolation protocols. Our program goal was to maximize the number of code blue responders completing this training within a 5-month time frame. Our primary objectives for learners were to improve the knowledge, skills, and behaviors required to participate in an airborne isolation code blue, with a secondary objective of improving learner confidence in the ability to provide high-quality care while maintaining provider safety. We believe our program provides a foundation for nursing and physician leadership at other hospitals, particularly in low-resource settings, to efficiently prepare staff for these high-risk events.

Methods

Development

Early in the COVID-19 pandemic, our Code Blue Committee identified compromised communication and significant delays in CPR initiation during airborne isolation code blue events despite the distribution of educational materials as outlined above. A COVID-19 subcommittee was formed to develop additional training and included a hospitalist, a critical care attending and fellow, and rapid response and critical care nursing leads. A traditional mock code format was deemed impractical as our simulation center was closed due to the pandemic, providers were stressed by a heavy clinical workload, and a high level of case complexity could overwhelm learners. We instead developed a low-resource talk-through walk-through approach. Three pilot exercises were conducted with 45 learners (internal medicine residents and unit nursing staff) to refine training. Initially, we found that learners struggled to focus on the key isolation-specific variables defined as our critical actions (see Appendix C). We then developed a script that supplied the core structure of advanced cardiac life support (ACLS)—rhythm identification, med dosing, and so on—without describing how to correctly perform the critical actions (communication with walkie-talkies, item transfer, etc.). We found that this format allowed trainees to focus on our key learning objectives.

Equipment/Environment

Materials included a CPR manikin to perform chest compressions, a pair of walkie-talkies, a defibrillator, a backboard, simulated medications (epinephrine and amiodarone), and lab tubes (Appendix D). The unit PPE bucket was an easily portable container kept on each unit, containing a backup supply of PPE (Appendix D). The PPE bucket was stored in the unit medication room and was brought to emergencies in the event PPE was not already available near the room or supplies were depleted. To preserve real PPE during the simulation, learners did not change their masks but donned reusable yellow contact isolation gowns to signal donning of airborne PPE. The unit's code blue crash cart with defibrillator was placed outside the room to create a more realistic environment but was not opened. The simulation was

Copyright © 2022 Collis et al. This is an open-access publication distributed under the terms of the Creative Commons Attribution license.

Table 1. Characteristics of Study Population

Role	Total Trained	Trained During Survey Period	Completed Survey	Response Rate
All participants	100	65	43	66%
Resident physician	29	16	16	100%
Acute care nurse	46	27	16	59%
ICU nurse	7	7	5	71%
STAT nurse	2	1	1	100%
Medical technician[a]	5	5	1	20%
Medical student[a]	5	3	2	67%
Respiratory therapist[a]	4	4	0	0%
Advanced practice provider	2	2	2	100%

[a]Other code blue responders.

typically conducted in an empty patient room on the target unit with the CPR manikin on the bed (Appendix D). During a census surge with no available empty patient rooms, simulations were conducted once in an empty physical therapy room and once in a staff breakroom with the CPR manikin on the table.

Personnel

Sessions were led outside the room by a single instructor, an internal medicine hospitalist or pulmonary critical care attending familiar with the airborne isolation code blue protocol. Inside the room, a standardized participant who acted as an ICU nurse provided prompts as needed to facilitate performance of the critical actions. The standardized participant was usually another program committee member, but when they were not available, this role was filled by an internal medicine resident (on three occasions) who was not a member of the code team. They received presession training to review the isolation code blue protocol and ICU nurse role in the simulation script, including prompts.

Implementation

From August to December 2020, we held 12 training sessions, each designed to accommodate two to four internal medicine residents and four to eight acute care, ICU, and STAT nurses. All learners were already in the hospital for clinical duty; therefore, sessions were limited to 30 minutes, with an option to stay later for additional questions or practice. Residents were preassigned to participate in groups of two within their first week on ward rotations during which they would serve as code blue leaders. Nurses were assigned by unit nurse managers to maximize the number of participants over time. Additional attendees, including respiratory therapists and advanced practice providers, were welcome to attend (Table 1). Sessions were announced the day prior, with a reminder 1 hour before training. Learners were encouraged to watch the hospital airborne isolation video training module (Appendix B) prior to the simulation but were not excluded if they did not do so. Some participants had already seen educational flyers or participated in real-life code blue events.

At the start of each session, learners were given a paper handout with the airborne isolation code blue protocol diagram (Appendix A) and code leader action priorities (Appendix E), as well as a 10-minute talk-through during which the instructor defined protocol roles and procedures and introduced the simulation approach. Each participant received a simulation script (Appendix F) and was assigned a role analogous to their code blue role in real clinical care. The simulation walk-through was 15 minutes long, beginning with the bedside nurse recognizing the patient was unresponsive and pulseless. The clinical scenario followed ACLS guidelines. Key scripted events facilitated practice of role assignment, room entry, communication via walkie-talkies, and transfer of items into and out of the room (Appendix C). The instructor observed these critical actions, which were repeated until learners were able to effectively perform them, as judged by the instructor. The simulation ended when teams successfully performed these actions at which time the patient achieved return of spontaneous circulation.

Assessment

A role-specific critical action checklist (Appendix C) was developed to reflect the key components of a successful airborne isolation code blue based on the hospital protocol. Critical actions were designed to be assessed from outside of the room with the door closed, as all components required interactions with the outside team that could be either visualized or heard. To ensure successful completion of critical actions by the inside team, the instructor observed inside team dynamics through glass doors or relied on reports from inside team participants and the ICU nurse standardized participant.

An anonymous postsession survey was shared with participants via a QR code link or paper (Appendix G). Participants were asked their role (resident, nurse, etc.) and previous airborne isolation

Copyright © 2022 Collis et al. This is an open-access publication distributed under the terms of the Creative Commons Attribution license.

code experience. On a 5-point Likert scale from strongly disagree to strongly agree, they rated their level of agreement with nine statements regarding pre- and posttraining knowledge, skill preparation, and confidence in quality of care and provider safety. A free-text option in the survey allowed open-ended feedback. Following the three initial pilot sessions, surveys were distributed from October to December 2020 to the 65 participants trained during that period. Statistical analysis was performed using Matlab R2020a with $\alpha = .05$. The chi-square test of independence was used to determine significance (pretraining, posttraining, and across groups). Recurrent themes in free-text responses were informally identified by grouping comments within a shared topic to qualitatively assess agreement. Investigational review board exemption was obtained from the University of Washington Medical Center (IRB ID STUDY00012136).

Debriefing

The debrief was led by the instructor, with input from the standardized participant ICU nurse. Participants reviewed the unique differences between airborne isolation and routine code blue events. They were asked to describe what made communication and item transfer in a two-team environment successful, what challenges arose in these areas or with the use of PPE, and how those challenges could be addressed. Learners were encouraged to raise questions or concerns and share their real-life experiences to inform protocol implementation or adjustments. The debrief was designed to be a minimum of 5 minutes to facilitate return to clinical duties; however, learners were given the option to remain longer and usually stayed for approximately 15 minutes.

Results

One hundred unique learners were trained during 12 training sessions from August to December 2020, including the 29 internal medicine residents who rotated on the acute care service at our hospital during this period. All learners successfully completed training and demonstrated the critical actions, as judged by the instructor in real time. Many learners initially struggled to complete the critical actions, particularly using the walkie-talkies to communicate. Learners were prompted to continue the simulation until all groups were able to complete all critical actions, as assessed by the instructor. During the nine sessions within the survey period of October-December 2020, 43 of 65 participants (66%) responded (see Table 1). Of these 43 participants, 24 (56%) reported prior exposure to any form of airborne isolation cardiac arrest training or materials (13 had read a flyer, three had participated in a nursing huddle, and eight had participated in a real-life airborne isolation code), while 19 (44%) had no prior experience.

Participants' self-assessment of pretraining protocol knowledge and skills is depicted in Table 2. Stratified by learner type, $\leq$25% of residents selected agree or strongly agree with their pretraining ability to identify who should enter the room ($n = 2$, 12%), where to find PPE ($n = 3$, 19%), ability to communicate effectively ($n = 2$, 12%), and ability to transfer items into and out of the room ($n = 4$, 25%; Table 2). Following training, there was a significant increase ($p < .001$) in the percentage of participants selecting agree or strongly agree for all statements. Improvement was independent of learner type (residents and nurses). There was no difference in posttraining scores between residents and nurses, except knowledge of where to acquire PPE (81% vs. 100%, respectively, $p < .05$).

Participants' confidence in their ability to provide high-quality care while maintaining safety for themselves and colleagues similarly improved after training. More than 95% of participants with prior airborne isolation code blue experience selected agree or strongly agree for all posttraining statements (Figure). The survey also allowed participants to provide free-text comments. Recurrent themes included benefits of the session design to help participants feel more prepared, requests for repeated sessions, and concern for communication challenges (Table 3).

Discussion

This talk-through walk-through scripted simulation training program allowed for efficient education and practice of airborne isolation code blue protocol for a large portion of resident and nursing staff in an urgent time frame. It was feasible to implement despite barriers such as physical distancing, staffing shortages, and financial strain that would limit high-fidelity simulation lab training. The program was designed and implemented by two physicians and one nurse, who spent no more than 1 hour per week conducting sessions. The program did not require the purchase of new equipment or technician support. Participants spent just 30 minutes away from work, requiring no special coverage. In the midst of an unprecedented global crisis, this simple design can serve as a framework to rapidly implement COVID-19 code blue training, particularly in low-resource communities at highest risk for outbreaks.[2]

Following simulation training, all participants reported increased self-assessed knowledge of and preparation for conducting an airborne isolation code, as well as increased confidence in quality of care and provider safety. It is notable that less than a quarter of residents reported an adequate baseline

Copyright © 2022 Collis et al. This is an open-access publication distributed under the terms of the Creative Commons Attribution license.

Table 2. Stratified Survey Results

Item	No. (%) Selecting Agree/Strongly Agree			
	Residents[a]	Nurses[b]	No Prior Experience[c]	Prior Experience[d]
Knowledge: I am able to identify:				
1. Which code team members should enter the room				
Pre	2 (12)	9 (41)	4 (21)	8 (33)
Post	15 (94)	20 (91)	16 (84)	24 (100)
p	<.001	<.001	<.001	<.001
2. When I need to wear airborne isolation PPE				
Pre	11 (69)	15 (68)	11 (58)	20 (83)
Post	16 (100)	21 (95)	18 (95)	24 (100)
p	<.05	<.05	<.01	<.05
3. Where to acquire appropriate PPE				
Pre	3 (19)	16 (73)	7 (37)	12 (50)
Post	13 (81)	22 (100)	15 (79)	24 (100)
P	<.001	<.01	<.01	<.001
Skills: I feel prepared to:				
4. Identify my role on the code team				
Pre	8 (50)	8 (36)	2 (11)	14 (58)
Post	16 (100)	21 (95)	18 (95)	24 (100)
P	<.01	<.001	<.001	<.001
5. Communicate effectively in two-team environment				
Pre	2 (12)	8 (36)	2 (11)	10 (42)
Post	15 (94)	16 (73)	12 (63)	24 (100)
P	<.001	<.05	<.001	<.001
6. Transfer items into and out of the code room				
Pre	4 (25)	11 (50)	3 (16)	14 (58)
Post	15 (94)	19 (86)	16 (84)	23 (96)
P	<.05	<.01	<.001	<.01
Safety: I am confident I will be able to:				
7. Provide quality care to my patient				
Pre	6 (38)	15 (68)	8 (42)	14 (58)
Post	13 (81)	21 (95)	16 (84)	23 (96)
P	<.05	<.05	<.01	<.01
8. Protect myself from high-risk exposure				
Pre	10 (62)	14 (64)	9 (47)	18 (75)
Post	15 (94)	20 (91)	16 (84)	24 (100)
P	<.05	<.05	<.05	<.01
9. Protect my colleagues from high-risk exposure				
Pre	9 (56)	12 (55)	7 (37)	16 (67)
Post	15 (94)	21 (95)	17 (89)	24 (100)
P	<.05	<.01	<.001	<.01

Abbreviation: PPE, personal protective equipment.
[a]Internal medicine residents, $n = 16$.
[b]Acute care, ICU, and STAT nurses; $n = 22$.
[c]$n= 19$.
[d]$n = 24$.

knowledge of essential team leader elements such as who should enter the room and how to communicate between inside and outside teams. Residents also rated a particularly low baseline knowledge of where to acquire appropriate PPE. This was notable because during the 2020-2021 winter surge, supply shortages limited the quantities of PPE available directly outside of patient rooms. Ensuring that residents knew where to locate additional emergency PPE or the unit PPE bucket was essential to facilitating their efficient and safe entry into the code room. These low baseline scores were likely related to frequent rotation of residents among hospitals and services, limiting reinforcement of airborne isolation practices. After training, residents showed robust improvement in these areas.

A simulation script allowed us to control for the large cognitive load of a traditional mock code, focusing learners on the critical actions related to airborne isolation code blue protocol. Free-text survey feedback supported this benefit of a scripted structure.

Our unique simulation design let us hold in situ sessions despite the significant resources and time typically required for mock codes. This allowed learners to locate the unit's equipment and PPE and to navigate the specific unit doors to safely enter, transfer items, and communicate during the code. When nonclinical environments were used, maintaining door type and function allowed us to proceed with these same benefits despite a surge in hospital census. In addition to being cost effective

Copyright © 2022 Collis et al. This is an open-access publication distributed under the terms of the Creative Commons Attribution license.

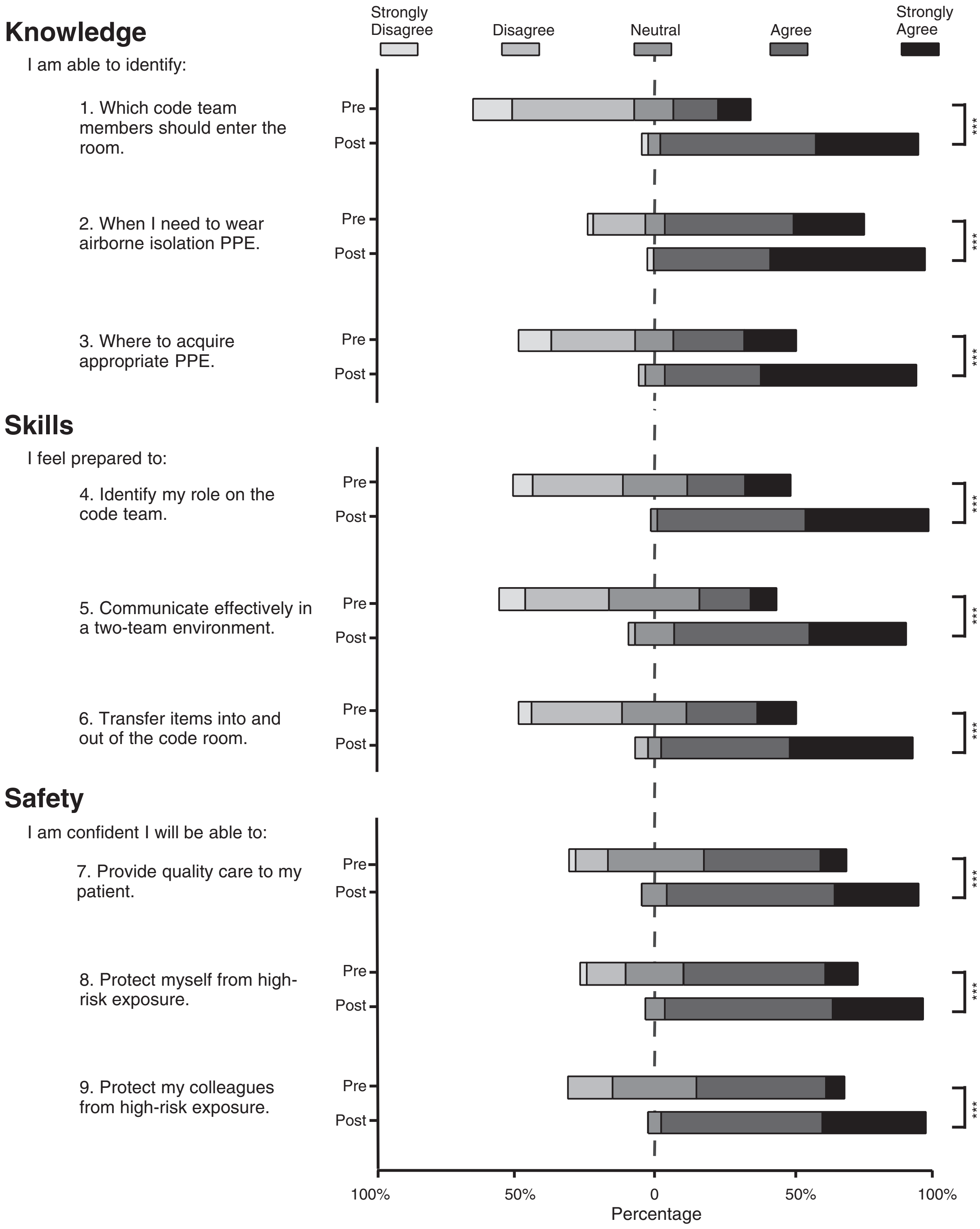

Figure. Percentage distribution of confidence in airborne isolation code blue knowledge, skills, and safety. Level of agreement was rated on a 5-point Likert scale (1 = *strongly disagree,* 5 = *strongly agree*) by participants pre- and postsimulation for questions 1-9. A retrospective pre-/postsurvey asked trainees to recall their level of agreement before and after the simulation. Survey questions 1-9 are listed on the *y*-axis. The *x*-axis (percentage) is aligned such that response 3 (*neutral*) is equally distributed on either side of 0. Statistical significance for the proportion of agree/strongly agree responses pre- versus posttraining is indicated by *** ($p < .001$). Abbreviation: PPE, personal protective equipment.

Copyright © 2022 Collis et al. This is an open-access publication distributed under the terms of the Creative Commons Attribution license.

Table 3. Open-Ended Survey Feedback

Theme	Representative Quotes[a]
Simulation design benefits	"Appreciated playing roles to get a feel for what it would really be like."—IM resident<br>"Easy to explain, hard to do. This is helpful practice."—IM resident<br>"Great to practice having a 'gate keeper' to keep crowd manageable and safe."—IM resident<br>"I appreciated the scripted simulation. It's a great time to review these mock code situations to keep staff safe and prepared for new types of situations."—acute care RN<br>"Good training simulating difficulties of COVID codes."—medical student
Repeated session requests	"Have a couple more to have more practice."—IM resident<br>"Keep offering classes so that everyone is ready."—acute care RN<br>"It was very good to practice. Continued periodic practice will be helpful."—acute care RN
Communication challenges	"Need to improve communication between two teams with the walkie-talkies."—IM resident<br>"On units where the outside team cannot see the inside team, there are issues with communicating with the walkie-talkies."—acute care RN<br>"Just concerned about communication between 2 teams."—acute care RN

Abbreviations: IM, internal medicine; RN, registered nurse.
[a]Participant role (IM resident, acute care RN, medical student) linked to quotes through the survey and identified above.

and resource efficient, our unique simulation format allowed us to stress-test our system and training program in a compressed time frame. Based on observations during early sessions, we refined our protocol and tailored our program to include just-in-time walkie-talkie training for residents. The talk-through element also allowed us to compensate for challenges to preeducation: Despite encouragement, almost half of our learners had not had any prior education. This likely reflects real-world conditions of heavy clinical workload during the COVID-19 crisis. While learners with pretraining had higher posttraining self-assessment and confidence, learners with no prior experience still showed significant improvement with our simulation format by obtaining preeducation (talk-through) and simulation within a single session.

Our learner-reported improvements are similar to those seen in prior cardiac arrest simulation literature,[26,27] and they add to a growing body of evidence supporting the utility of simulation programs to prepare for COVID-19 cardiac arrest and other rare resuscitation events.[13-20,28] Unlike previous work, however, our talk-through walk-through structure and scripted simulation is far simpler to implement in time-, personnel-, and resource-constrained settings such as those with current outbreaks due to low vaccination rates. While we cannot directly compare our outcomes to a traditional high-fidelity simulation structure, some of our highest-risk communities are less likely to have a dedicated simulation lab. Nursing and physician leadership at other hospitals can easily adopt our protocol and materials or customize the simulation script to reflect their own airborne protocol. Additionally, this approach is easily adapted to other airborne isolation code events, such as patients with *M. tuberculosis* or disseminated varicella infections, and can be used to rapidly implement other changes to emergency response.

Our work has several limitations. This was a single-center program, and although it is easily adaptable to other environments, its results may not be generalizable. Our simulation design was heavily dictated by the resource, time, and personnel demands of an ongoing pandemic. Similarly, these barriers prevented the use of pre- and posttraining objective assessments to quantitatively document improvement in performance of large numbers of learners in diverse roles, due to clinical need and overall program goals. Therefore, we utilized self-assessment in a retrospective survey tool, which has inherent limitations of bias. While this was an appropriate approach to address our secondary objectives of improved learner confidence in ability to provide quality care and maintain provider safety, future work during less strained situations would benefit from more rigorous skill-assessment methods to optimize the program. As resources become more accessible, future work could also include incorporating formal presession education.

Overall, our simulation program was successful at efficiently educating resident and nursing staff regarding airborne isolation cardiac arrest using minimal resources and personnel. It increased the number of learners exposed to airborne isolation code blue protocol and improved self-assessed knowledge, skills, and confidence to participate in real-life airborne isolation cardiac arrest.

Appendices

A. Protocol Diagram.docx
B. Training Video.mp4
C. Simulation Case Template.docx
D. Simulation Images.pdf
E. Action Priorities.docx

Copyright © 2022 Collis et al. This is an open-access publication distributed under the terms of the Creative Commons Attribution license.

F. Simulation Script.docx

G. Survey.docx

All appendices are peer reviewed as integral parts of the Original Publication.

Alexandra C. Collis, MD: Clinical Instructor, Division of General Internal Medicine, University of Washington Medical Center; ORCID: https://orcid.org/0000-0002-6352-0092

Andrew P. Wescott, MD, PhD: Second-Year Internal Medicine Resident, University of Washington Medical Center

Sheryl Greco, MN, RN: Critical Care/Cardiac Clinical Nurse Specialist, University of Washington Medical Center

Nicole Solvang, RN, BSN, CCRN: Resource Team Assistant Nurse Manager for Critical Care, STAT RNs and Vascular Access, University of Washington Medical Center

Joshua Lee, MD: Fellow, Pulmonary and Critical Care, University of Washington Medical Center

Amy E. Morris, MD: Associate Professor, Division of Pulmonary, Critical Care, and Sleep Medicine, University of Washington Medical Center

Disclosures
None to report.

Funding/Support
None to report.

Ethical Approval
The University of Washington Medical Center Institutional Review Board deemed further review of this project not necessary.

References

1. Lopez Bernal J, Andrews N, Gower C, et al. Effectiveness of Covid-19 vaccines against the B.1.617.2 (delta) variant. *N Engl J Med*. 2021;385(7):585-594. https://doi.org/10.1056/NEJMoa2108891
2. Murthy BP, Sterrett N, Weller D, et al. Disparities in COVID-19 vaccination coverage between urban and rural counties—United States, December 14, 2020–April 10, 2021. *MMWR Morb Mortal Wkly Rep*. 2021;70(20):759-764. https://doi.org/10.15585/mmwr.mm7020e3
3. DeFilippis EM, Ranard LS, Berg DD. Cardiopulmonary resuscitation during the COVID-19 pandemic: a view from trainees on the front line. *Circulation*. 2020;141(23):1833-1835. https://doi.org/10.1161/CIRCULATIONAHA.120.047260
4. Scquizzato T, Olasveengen TM, Ristagno G, Semeraro F. The other side of novel coronavirus outbreak: fear of performing cardiopulmonary resuscitation. *Resuscitation*. 2020;150:92-93. https://doi.org/10.1016/j.resuscitation.2020.03.019
5. Couper K, Taylor-Phillips S, Grove A, et al. COVID-19 in cardiac arrest and infection risk to rescuers: a systematic review. *Resuscitation*. 2020;151:59-66. https://doi.org/10.1016/j.resuscitation.2020.04.022
6. Hassager C, Price S, Huber K. Cardiac arrest in the COVID-19 era. *Eur Heart J Acute Cardiovasc Care*. 2020;9(3):239-240. https://doi.org/10.1177/2048872620922789
7. Thorne CJ, Ainsworth M. COVID-19 resuscitation guidelines: a blanket rule for everyone? *Resuscitation*. 2020;153:217-218. https://doi.org/10.1016/j.resuscitation.2020.06.013
8. Ramzy M, Montrief T, Gottlieb M, Brady WJ, Singh M, Long B. COVID-19 cardiac arrest management: a review for emergency clinicians. *Am J Emerg Med*. 2020;38(12):2693-2702. https://doi.org/10.1016/j.ajem.2020.08.011
9. Cheruku S, Dave S, Goff K, et al. Cardiopulmonary resuscitation in intensive care unit patients with coronavirus disease 2019. *J Cardiothorac Vasc Anesth*. 2020;34(10):2595-2603. https://doi.org/10.1053/j.jvca.2020.06.008
10. Song W, Liu Y, Ouyang Y, et al. Recommendations on cardiopulmonary resuscitation strategy and procedure for novel coronavirus pneumonia. *Resuscitation*. 2020;152:52-55. https://doi.org/10.1016/j.resuscitation.2020.03.023
11. Wax RS, Christian MD. Practical recommendations for critical care and anesthesiology teams caring for novel coronavirus (2019-nCoV) patients. *Can J Anaesth*. 2020;67(5):568-576. https://doi.org/10.1007/s12630-020-01591-x
12. Edelson DP, Sasson C, Chan PS, et al.; American Heart Association ECC Interim COVID Guidance Authors. Interim guidance for basic and advanced life support in adults, children, and neonates with suspected or confirmed COVID-19: from the Emergency Cardiovascular Care Committee and Get With the Guidelines-Resuscitation Adult and Pediatric Task Forces of the American Heart Association. *Circulation*. 2020;141(25):e933-e943. https://doi.org/10.1161/CIRCULATIONAHA.120.047463
13. McGaghie WC, Issenberg SB, Cohen ER, Barsuk JH, Wayne DB. Does simulation-based medical education with deliberate practice yield better results than traditional clinical education? A meta-analytic comparative review of the evidence. *Acad Med*. 2011;86(6):706-711. https://doi.org/10.1097/ACM.0b013e318217e119
14. Lee A, Sheen JJ, Richards S. Intrapartum maternal cardiac arrest: a simulation case for multidisciplinary providers. *MedEdPORTAL*. 2018;14:10768. https://doi.org/10.15766/mep_2374-8265.10768
15. Halverson B, Malkin M, Lenart J, Vadi M. Simulation case: cardiac arrest after carbon dioxide (CO2) embolism. *MedEdPORTAL*.

Copyright © 2022 Collis et al. This is an open-access publication distributed under the terms of the Creative Commons Attribution license.

2016;12:10327. https://doi.org/10.15766/mep_2374-8265.10327

16. Conlon L, Johnson N. Simulation case: hanging/cardiac arrest. *MedEdPORTAL*. 2014;10:9923. https://doi.org/10.15766/mep_2374-8265.9923
17. Sinha T, Stinehart K, Moorer C, Spitzer C. Cardiopulmonary arrest and resuscitation in the prone patient: an adult simulation case for internal medicine residents. *MedEdPORTAL*. 2021;17:11081. https://doi.org/10.15766/mep_2374-8265.11081
18. Andreae MH, Dudak A, Cherian V, et al. Healthcare simulation to prepare for the COVID-19 pandemic. *J Clin Anesth*. 2020;66:109928. https://doi.org/10.1016/j.jclinane.2020.109928
19. Dieckmann P, Torgeirsen K, Qvindesland SA, Thomas L, Bushell V, Ersdal HL. The use of simulation to prepare and improve responses to infectious disease outbreaks like COVID-19: practical tips and resources from Norway, Denmark, and the UK. *Adv Simul (Lond)*. 2020;5:3. https://doi.org/10.1186/s41077-020-00121-5
20. Malysz M, Dabrowski M, Böttiger BW, et al. Resuscitation of the patient with suspected/confirmed COVID-19 when wearing personal protective equipment: a randomized multicenter crossover simulation trial. *Cardiol J*. 2020;27(5):497-506. https://doi.org/10.5603/CJ.a2020.0068
21. Maran NJ, Glavin RJ. Low- to high-fidelity simulation—a continuum of medical education? *Med Educ*. 2003;37(s1):22-28. https://doi.org/10.1046/j.1365-2923.37.s1.9.x
22. National Research Council. *Learning, Remembering, Believing: Enhancing Human Performance*. National Academies Press; 1994.
23. Wenlock RD, Arnold A, Patel H, Kirtchuk D. Low-fidelity simulation of medical emergency and cardiac arrest responses in a suspected COVID-19 patient—an interim report. *Clin Med (Lond)*. 2020;20(4):e66-e71. https://doi.org/10.7861/clinmed.2020-0142
24. Chung SP, Cho J, Park YS, et al. Effects of script-based role play in cardiopulmonary resuscitation team training. *Emerg Med J*. 2011;28(8):690-694. https://doi.org/10.1136/emj.2009.090605
25. Lee M, Chen S, Tosif S, Graham J. "Talk-through walk-through"—a simulation approach adapted during preparation for COVID-19. *Simul Healthc*. Published online 2020. https://doi.org/10.1097/SIH.0000000000000525
26. Stefan MS, Belforti RK, Langlois G, Rothberg MB. A simulation-based program to train medical residents to lead and perform advanced cardiovascular life support. *Hosp Pract (*1995*)*. 2011;39(4):63-69. https://doi.org/10.3810/hp.2011.10.923
27. Mundell WC, Kennedy CC, Szostek JH, Cook DA. Simulation technology for resuscitation training: a systematic review and meta-analysis. *Resuscitation*. 2013;84(9):1174-1183. https://doi.org/10.1016/j.resuscitation.2013.04.016
28. Wayne DB, Butter J, Siddall VJ, et al. Simulation-based training of internal medicine residents in advanced cardiac life support protocols: a randomized trial. *Teach Learn Med*. 2005;17(3):210-216. https://doi.org/10.1207/s15328015tlm1703_3

Received: September 14, 2021
Accepted: October 13, 2021
Published: January 14, 2022

Copyright © 2022 Collis et al. This is an open-access publication distributed under the terms of the Creative Commons Attribution license.
